# A Maladaptive Role for EP4 Receptors in Mouse Mesangial Cells

**DOI:** 10.1371/journal.pone.0104091

**Published:** 2014-08-14

**Authors:** Guang-xia Yang, Yu-yin Xu, Ya-ping Fan, Jing Wang, Xiao-lan Chen, Yi-de Zhang, Jian-hua Wu

**Affiliations:** 1 Department of Nephrology, Affiliated Hospital of Nantong university, Nantong, Jiangsu, China; 2 Department of Rheumatology, Affiliated Hospital of Jiangnan University (Wuxi 4th People's Hospital), Wuxi, Jiangsu, China; 3 Shanghai Jiaotong University, School of Medicine, Shanghai, China; University of Rochester Medical Center, United States of America

## Abstract

Roles of the prostaglandin E2 E-prostanoid 4 receptor (EP4) on extracellular matrix (ECM) accumulation induced by TGF-β1 in mouse glomerular mesangial cells (GMCs) remain unknown. Previously, we have identified that TGF-β1 stimulates the expression of FN and Col I in mouse GMCs. Here we asked whether stimulation of EP4 receptors would exacerbate renal fibrosis associated with enhanced glomerular ECM accumulation. We generated EP4^Flox/Flox^ and EP4^+/−^ mice, cultured primary WT, EP4^Flox/Flox^ and EP4^+/−^ GMCs, AD-EP4 transfected WT GMCs (EP4 overexpression) and AD-Cre transfected EP4^Flox/Flox^ GMCs (EP4 deleted). We found that TGF-β1-induced cAMP and PGE2 synthesis decreased in EP4 deleted GMCs and increased in EP4 overexpressed GMCs. Elevated EP4 expression in GMCs augmented the coupling of TGF-β1 to FN, Col I expression and COX2/PGE2 signaling, while TGF-β1 induced FN, Col I expression and COX2/PGE2 signaling were down-regulated in EP4 deficiency GMCs. 8 weeks after 5/6 nephrectomy (Nx), WT and EP4^+/−^ mice exhibited markedly increased accumulation of ECM compared with sham-operated controls. Albuminuria, blood urea nitrogen and creatinine (BUN and Cr) concentrations were significantly increased in WT mice as compared to those of EP4^+/−^ mice. Urine osmotic pressure was dramatically decreased after 5/6 Nx surgery in WT mice as compared to EP4^+/−^ mice. The pathological changes in kidney of EP4^+/−^ mice was markedly alleviated compared with WT mice. Immunohistochemical analysis showed significant reductions of Col I and FN in the kidney of EP4^+/−^ mice compared with WT mice. Collectively, this investigation established EP4 as a potent mediator of the pro-TGF-β1 activities elicited by COX2/PGE2 in mice GMCs. Our findings suggested that prostaglandin E2, acting via EP4 receptors contributed to accumulation of ECM in GMCs and promoted renal fibrosis.

## Introduction

Renal fibrosis is the underlying pathological alteration and the common way of almost all progressive kidney diseases. Fibrosis is considered a uniform process defined as exaggerated deposition of nonfunctional scar tissue comprising extracellular matrix (ECM) and fibroblasts [1]. The ECM is mainly produced by mesangial cells (MCs) and contains collagens type I, IV and V, laminin A, B1 and B2, fibronectin, heparan sulfate and chondroitin sulfate proteoglycans, entactin, nidogen and etc. ECM is the major factor of mesangial expansion as seen in many glomerular diseases associated with increased synthesis in the MCs [2]. Thus, MCs play a critical role in initiation of glomerular inflammation and progression to chronic kidney disease. TGF-β plays an essential role in MCs hypertrophy associated with diabetes and other glomerulopathies [3] through CTGF-mediated mechanism [4]. Enhanced expression of the TGF-β1 gene is one of the most permanent molecular changes causing pathological tissue fibrosis [5].

Prostaglandins (PGs), mainly PGE2, play important roles in renal hemodynamics, renin release and salt and water homeostasis. PGE2 is synthesized from arachidonic acid. Briefly, arachidonic acid is converted to an unstable intermediate PGH2 by cyclooxygenase (COX), then PGH2 converted to PGE2 by prostaglandin E synthase (PGES) [6]–[8]. Two isoforms of COX exist in mammals, “constitutive” COX-1 and inflammatory-mediated and glucocorticoid-sensitive COX-2. COX-1 is expressed in mammalian kidney at vasculature, glomerular mesangial cells, and the collecting duct. Subsequent studies have documented COX-2 expression in macula densa (MD) and cortical thick ascending limb (cTAL) and medullary interstitial cells in kidney of mouse, rat, rabbit, dog, and human, as well as lower levels of expression in podocytes and renal arterioles [9]. COX-2 participates in a number of pathological processes in immune-mediated renal diseases, and it is well known that protein kinase B (AKT) may act through different transcription factors in the regulation of the COX-2 promoter. The physiological effects of PGE2 are mediated through prostaglandin E receptors (EP receptors). Four subtypes of EP receptors (EP1 to EP4) are currently known. Stimulation of the EP1 receptor results in activation of phosphatidylinositol (PI) hydrolysis and elevation of intracellular Ca^2+^ concentration [10]. EP2 and EP4 receptors couple to Gs, and activation of these receptors results in stimulation of adenylyl cyclase and increases intracellular cAMP [11]. The major signaling pathway described for the EP3 receptor is mediated by Gi and leads to a reduction in intracellular cAMP levels [12]. The EP4 receptor is coupled to a Gs protein, it’s effect on cAMP formation is weaker than that of EP2. Furthermore, the EP4 receptor is able to activate the phosphoinositide 3-kinase-dependent pathway, then activate MAPK (mitogen-activated protein kinase) signalling [13], [14]. Studies on podocyte have demonstrated that prostaglandin E2 acting via EP4 receptors contributes to the early deterioration of the glomerular filtration barrier (GFB) in vivo, which highlights the pleiotropic nature of the intrarenal actions of the prostanoid family [15].

Positive or negative feedback regarding the EP4 expression may occur in the fibrosis progress of various renal diseases, and such feedback could have relevant impacts on the development of kidney diseases. Our previous study showed that TGF-β1 could increase both COX-2 and PGE2 by means of stimulating GMCs, promote CTGF expression as well.

The purpose of the present work was to research the impact of EP4 expression on TGF-β1 induced ECM accumulation of mouse GMCs, and investigate renal pathological changes in EP4^+/−^ mice after 5/6 Nx to explore the possible mechanisms involved in kidney fibrosis induced by EP4 activation.

## Materials and Methods

### Ethics Statement

All animals were available by the Animal Experimentation Committee of Beijing University Health Science Center according to SPF environment. Animal protocols for these studies were approved by the Beijing University Animal Care and Use Committee. All mice were fed with standard animal food and kept in an air-conditioned environment. The animals were allowed free access to water and food before and after surgery. The animals were euthanized with an overdose of sodium thiopental.

### Experimental Animals

25 WT (wild-type), 25 EP4 heterozygotes (EP4^+/−^) and 5 EP4^Flox/Flox^ (EP4^Flox/Flox^ mice have loxP sites flanking exon 2 of the EP4 gene, making it a conditional knock-out EP4 gene sequence between LoxP sites; can be cut out by adenoviral cre) male mice aged from 8 to 12 weeks, with a gene background of C57/BL6 were kept in 11 cages, 5 per cage. All mice were fed with animal food and kept in an air-conditioned environment, light/dark 12 h cycle. The mice were euthanized with an overdose of sodium thiopental.

### Cell Culture

Kidneys from 8 to 12 week old male WT, EP4^+/−^ or EP4^Flox/Flox^ mice were obtained from Animal Center, Beijing University. Glomeruli were purified from renal cortex tissue, then the glomeruli suspension was digested for 40 minutes at 37°C with 0.1% type I collagenase. Glomeruli were then collected through 70 µm and 40 µm stainless steel sieves. The digested samples were then centrifuged at 1000 rpm for 5 minutes, and the precipitate was resuspended in growth medium (Dulbecco’s modified Eagle’s medium supplemented with 20% fetal bovine serum [Gibco, Invitrogen]). The glomeruli were cultured at 37°C in a humidified incubator containing 5% CO_2_. The primary mice GMCs at passages 8 to 10 were used and treated differently according to experimental protocols. GMCs were divided into 4 groups: 1)WT group; 2)WT+TGF-β1 group; 3)EP4^+/−^group; 4)EP4^+/−^+TGF-β1 group. WT GMCs were transfected with AD-EP4 (moi = 5) or AD-GFP (moi = 5), cells were divided into 4 groups: 1)WT+AD-GFP group; 2)WT+AD-GFP+TGF-β1 group; 3)WT+AD-EP4 group; 4)WT+AD-EP4+TGF-β1 group. EP4^Flox/Flox^ GMCs were transfected with AD-GFP (moi = 10) and AD-Cre (moi = 10) also divided into 4 groups: 1)EP4^Flox/Flox^ + AD-GFP group; 2)EP4^Flox/Flox^ + AD-GFP+TGF-β1 group; 3)EP4^Flox/Flox^ + AD-Cre group; 4)EP4^Flox/Flox^ + AD-Cre+TGF-β1 group. Before the experiments, the cells were incubated without fetal bovine serum for 24 h. Based on the previous experimental results, the best reaction time point and the optimal dose of TGF-β1 was 10 ng/ml at 24 h. Each individual experiment was repeated at least 3 times with different cell preparations.

### Adenoviral Constructs and Infection of Cultured mice mesangial cells

The AD-EP4, AD-GFP and AD-Cre were generated by the Shanghai GenePharma Co. Ltd. The EP4 coding sequence was sub-cloned out of its host plasmid (pCMV-Sport6) into pcDNA3 using EcoRI/XhoI restriction enzyme sites. An N-terminal 2HA tag was introduced by PCR-based cloning using the following primers: forward- gatccattatccatatgacgtcccagactctgcctatccatatgacgtcccagactctgccg; reverse- aattcggcagagtctgggacgtcatatggataggcagagtctgggacgtcatatggatacatg.

Linearized recombinant adenoviral plasmid was transfected into AD-293 cells to obtain a primary viral stock, which was amplified and purified. For optimization of infection conditions, differentiated mouse GMCs were infected with EP4 adenovirus at a 5 multiplicity of infection (MOI) for 72 hours. The MOI of AD-Cre was 10. Expression of EP4 in infected WT GMCs was examined by Western blot.

### Measurement of cAMP Levels

PGE2-mediated activation of EP4 receptors results in the production of cAMP. EP4^+/−^ mouse GMCs are EP4 semi-knockout cells. To minimize the expression of EP4, we generated EP4^Flox/Flox^ GMCs and transfected with AD-Cre to knockout the expression of EP4. In order to confirm the expression of EP4, cell supernatant cAMP levels were measured by immunoassay. WT GMCs were transfected with AD-GFP and AD-EP4 (MOI = 5), EP4^Flox/Flox^ GMCs were transfected with AD-GFP and AD-Cre (MOI = 10), all cells were treated with 10 ng/L TGF-β1 for 24 h. Cell supernatant were collected. The amount of cAMP in the cell supernatant samples was quantified using an Alpha screen cAMP Assay Kit (Perkin Elmer, Massachusetts, USA), according to the manufacturer’s instructions, and read by an EnVision Multilabel Plate Reader.

### RNA Extraction and Real-Time PCR

Total RNA of cells from different groups was isolated by Trizol reagent (Life Technologies). Total RNA was transferred to cDNA by use of the TaqMan Reverse Transcription Reagents kit (Applied Biosystems) according to the manufacturer’s protocol. Real-time PCR was performed with use of iCycler with the SYBR Green I probe (Bio-Rad, Hercules, CA). Each sample was analyzed in triplicate and normalized to the level of β-actin mRNA. The FN PCR protocol was 95°C 5 min→(95°C 30 s→56°C 30 s→72°C 30 s)×35→72°C 10 min. The Col Ìβ-actin PCR protocol was 95°C 5 min→(95°C 30 s→57°C 30 s→72°C 30 s)×35→ 72°C 10 min. The primer sequences are in Table 1. PCR products were validated by electrophoresis on 2% agarose gel.

**Table 1:** Primers for real-time PCR analysis.

	Chain	Sequence (5′-3′)	Product (bp)
β-actin	FP	TTTAATTTCACGCACGATTTC	150
	RP	CCCATCTATGAGGGTTACGC	
FN	FP	AATGGAAAAGGGGAATGGAC	244
	RP	CTCGGTTGTCCTTCTTGCTC	
Col I	FP	GAGCGGAGAGTACTGGATCG	142
	RP	GTTCGGGCTGATGTACCAGT	
COX1	FP	GTGGCTATTTCCTGCAGCTC	209
	RP	CAGTGCCTCAACCCCATAGT	
COX2	FP	AGAAGGAAATGGCTGCAGAA	194
	RP	GCTCGGCTTCCAGTATTGAG	
mPGES-1	FP	CGCGGTGGCTGTCATCA	205
	RP	AGGGTTGGGTCCCAGGAAT	
mPGES-2	FP	GACCCTGTACCAGTACAAGAC	277
	RP	GGCCTTCATGGGTGGGTA AT	
cPGES	FP	ATGCAGCCTGCTTCTGCA	483
	RP	TTACTCCAGATCTGGCAT	

### Western blot analyses

Immunoprecipitation cell lysis buffer was added to the wells, and the plate was put on ice for 30 minutes, then cells treated as described above were scraped, and cell lysate was removed to 1.5 ml EP tubes and spun for 15 minutes. The Protein concentrations in supernatant were determined by BCA assay (Pierce, Rockford, USA). Samples were diluted in the loading buffer, and then boiled for 5 minutes. Then 130 µg of each sample was separated on a 10% sodium dodecyl sulfate–polyacrylamide gel electrophoresis (SDS-PAGE) and transferred to a PVDF membrane for 2 h, and then blocked at room temperature for 1 h in 5% (w/v) non-fat dry milk. The PVDF membrane were incubated with primary antibodies (mouse anti-FN, mouse anti-Col-I, mouse anti-cox1, mouse anti-cox2, mouse anti-mPGES1, mouse anti-mPGES2, mouse anti-cPGES, 1∶1,000; Rockland immunochemical) at 4°C overnight. The membrane was washed with Tris Buffered Saline with Tween (TBST), incubated with DyLight 800-labeled antibody to mouse IgG (1∶5000) for 2 h, and the membrane was scanned by the Bio-Rad Imaging System for semiquantitative analysis.

### PGE2 analysis

Cell supernatant PGE2 levels were measured with an Alpha screen PGE_2_ Assay Kit (Perkin Elmer, Massachusetts, USA). Cells were divided into 12 groups: WT, EP4^+/−^, WT+AD-GFP, WT+AD-EP4, EP4^Flox/Flox^ + AD-GFP and EP4^Flox/Flox^ + AD-Cre treated with or without TGF-β1 for 24 h. For PGE2 determination, supernatant was collected and PGE2 levels were measured according to the supplier’s instructions.

### 5/6 Nephrectomy

Adult male WT and EP4^+/−^ C57BL/6 mice underwent 5/6 nephrectomy or a sham operation (10 mice in each group). Under sodium thiopental-induced anesthesia, age-matched male and female mice (8 to 10 weeks) underwent renal ablation via removal of five sixths of total renal mass. The surgical procedure was carried out by resecting the right kidney and cauterizing the upper and lower poles of the left kidney. Once the left side was stitched, the dorsal incision was closed using stainless steel wound clips before the mouse was placed in recovery. Control mice underwent sham operations without the removal of any renal mass.

### Sample collection

Prior to sacrifice, WT sham, WT 5/6 Nx, EP4^+/−^ sham and EP4^+/−^5/6 Nx mice (n = 10, respectively) were placed individually in metabolic cages for 24-h urine collection at 4, 6 and 8 weeks after 5/6 Nx, respectively. Urine samples were centrifuged at 1,000×g for 10 min at 20°C and the supernatants were stored at −20°C until analysis. Prior to sacrifice, the mice were then anaesthetized by sodium thiopental for collection of blood and kidneys. The blood (about 1.5 ml) was collected from the abdominal aorta in heparinized tubes and was centrifuged at 900 g for 15 min at 4°C to obtain plasma. The plasma obtained was stored at −80°C for a week or less pending analyses. The kidneys were quickly removed and either frozen immediately in liquid nitrogen or fixed with 4% buffered formalin. At the end, animals were killed by overdose of sodium thiopental.

### Biochemical analysis

Albuminuria was determined by the Mouse Albumin ELISA kit (Shibayagi Co., Ltd., Gunma, Japan). Blood creatinine (Cr) concentrations were measured by Jaffe's method using the Creatinine-Test-Wako (Wako Pure Chemical Industries, Ltd., Osaka, Japan). BUN was measured by Kinetic UV assay for urea/urea nitrogen kit (Roche Diagnostics GmbH, Mannheim, Germany). Urinary osmolality was measured by Freezing-Point Osmometer (Loser, German).

### Renal histological analysis

Eight weeks after 5/6 nephrectomy or sham surgery, the animals were euthanized with an overdose of sodium thiopental. and perfused via the left ventricle with phosphate-buffered saline (0.1 M PBS, pH 7.4), followed by a fixative solution of formaldehyde (4%); the kidney was removed, cleaned of connective tissue and embedded in paraffin. Three micrometer-thick sections were processed with periodic acid-Schiff (PAS) staining, and the glomeruli were photographed for later analysis. Images were captured with color video camera (VKC150; Hitachi, Tokyo, Japan) connected to a microscope (AX70; Olympus, Center Valley, PA) and analyzed with a specific image program (2100 Leica EWS; Leica, Wetzlar, Germany) by a person blinded to the experimental groups.

### Immunohistochemistry

Matrix protein expression (collagen I and fibronectin) was analyzed using immunohistochemical staining methods. For antibody incubation, 3µm thick paraffin sections were mounted on poly-L-lysine coated glass slides. Polyclonal rabbit antibodies against collagen type I and fibronectin were used. Detection of the bound antibodies was performed using a HRP second antibody and using 3, 3-diaminobenzidine (DAB) as selenium organic reagent, according to the manufacturer's instructions (Abcam, England). Control experiments were performed omitting the first antibody and using phosphate-buffered saline instead. The integrated optical density of the positive material in each glomerulus and the glomerular area were measured by the morphological analysis system, the ratio of which showed the relative content of FN and Col I in the renal glomeruli.

### Statistical Analysis

All values are expressed as mean±SEM. Data were analyzed by Student t test (paired groups) or 2-way ANOVA, followed by Bonferroni’s post test correction (multigroup comparisons). P<0.05 was considered statistically significant.

## Results

### EP4^+/−^ and EP4^Flox/Flox^ mice identified

**Figure 1:**
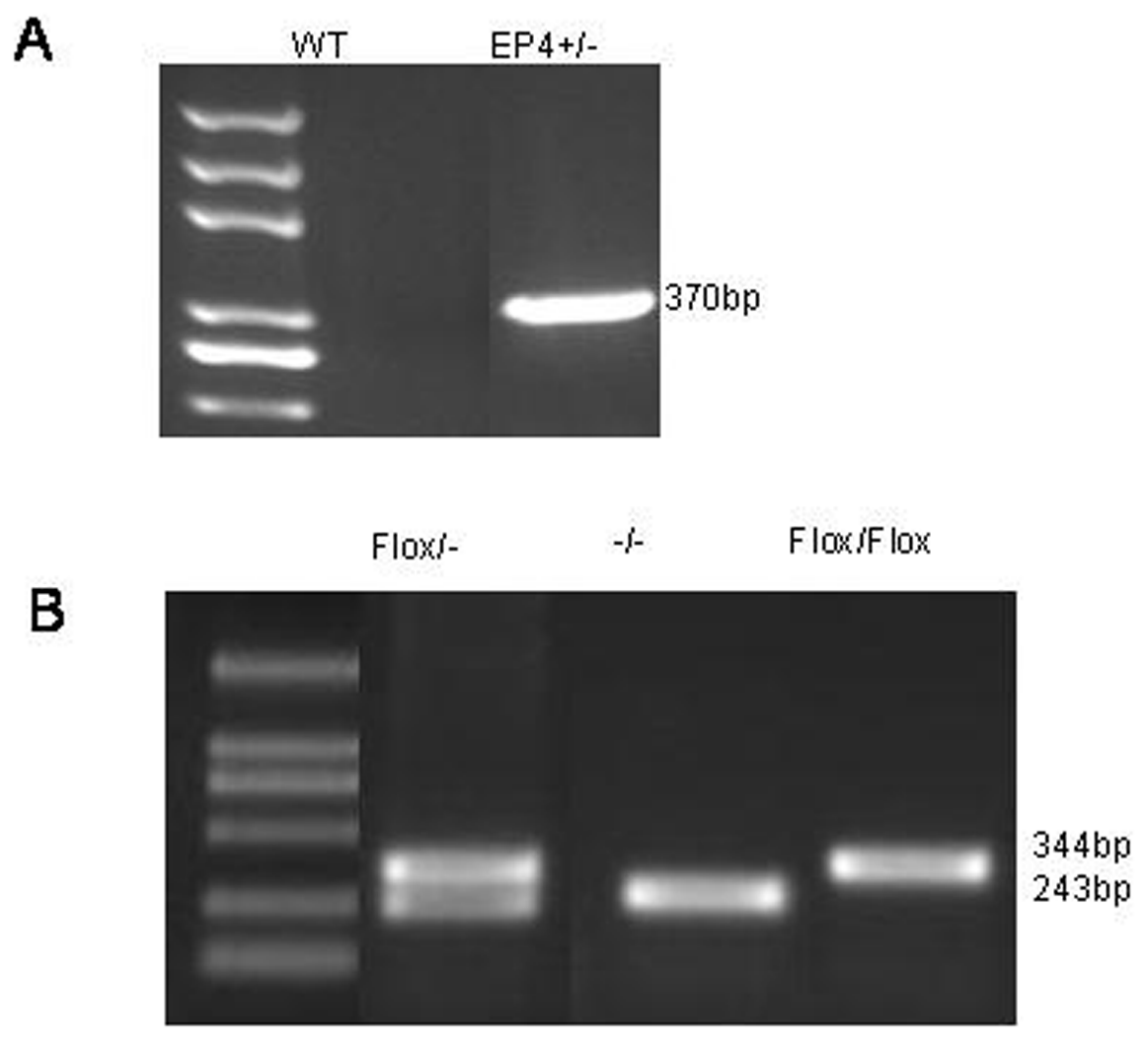
EP4+/− and EP4Flox/Flox mice identified. A. The exon 2 region of WT mice was not knockout and gene length was too long to have PCR product. The exon 2 region of EP4^+/−^ mice was knockout and PCR product was 370 bp. B. PCR product of EP4^Flox/Flox^ mice was 344 bp and WT mice was 243 bp.

Total RNA was extracted from the GMCs of WT, EP4^+/−^ and EP4^Flox/Flox^ mice. EP4^+/−^ mice were identified by PCR (Fig. 1A). The primers were designed on the two ends of exon 2. The exon 2 region of WT mice was not knocked out and gene length was too long to have PCR product. The exon 2 region of EP4^+/−^ mice was deleted and PCR product was 370 bp. EP4^Flox/Flox^ mice were also identified by PCR (Fig. 1B). PCR products of WT and EP4^Flox/Flox^ mice were 243 bp and 344 bp, respectively.

### Expression of EP4 in different treated mouse GMCs

**Figure 2:**
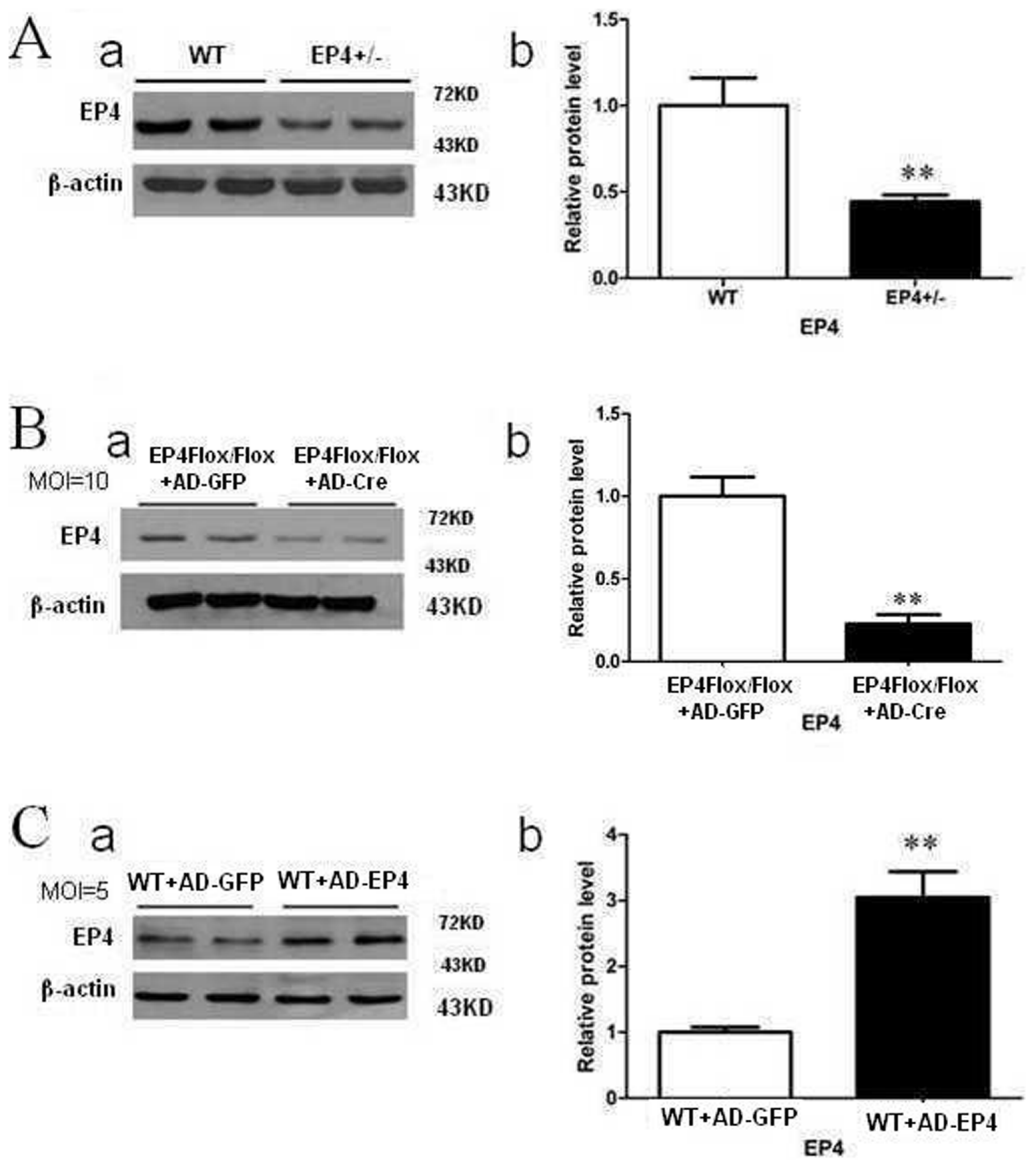
Expression of EP4 in different treated mouse GMCs. A: Expression of EP4 in EP4^+/−^ mouse GMCs was detected by western blot. B: Expression of EP4 protein in EP4^Flox/Flox^ mouse GMCs infected with AD-Cre detected by western blot. **C**: Expression of EP4 protein in WT GMCs infected with AD-EP4 detected by western blot. (β-actin was used as an internal control. a: Western blot; b: Quantification of EP4 expression is achieved using densitometric values normalized to β-actin levels, the means and error bars are the result of biological replicates.

We isolated and cultured primary GMCs from WT, EP4^Flox/Flox^ and EP4^+/−^ mice. As shown in Fig. 2A, protein obtained from primary cultured GMCs of EP4^+/−^ mice showed a statistically significant 50% reduction in EP4 receptor expression as compared with WT mice. EP4^Flox/Flox^ GMCs infected with AD-Cre (moi = 10) had significantly lower EP4 expression than EP4^Flox/Flox^ GMCs infected with AD-GFP (P<0.05, Fig. 2B). In contrast, WT GMCs infected with AD-EP4 (moi = 5) had significantly higher EP4 expression than WT GMCs infected with AD-GFP (P<0.05, Fig. 2C).

### EP4 deficiency reduced cAMP secretion

**Figure 3:**
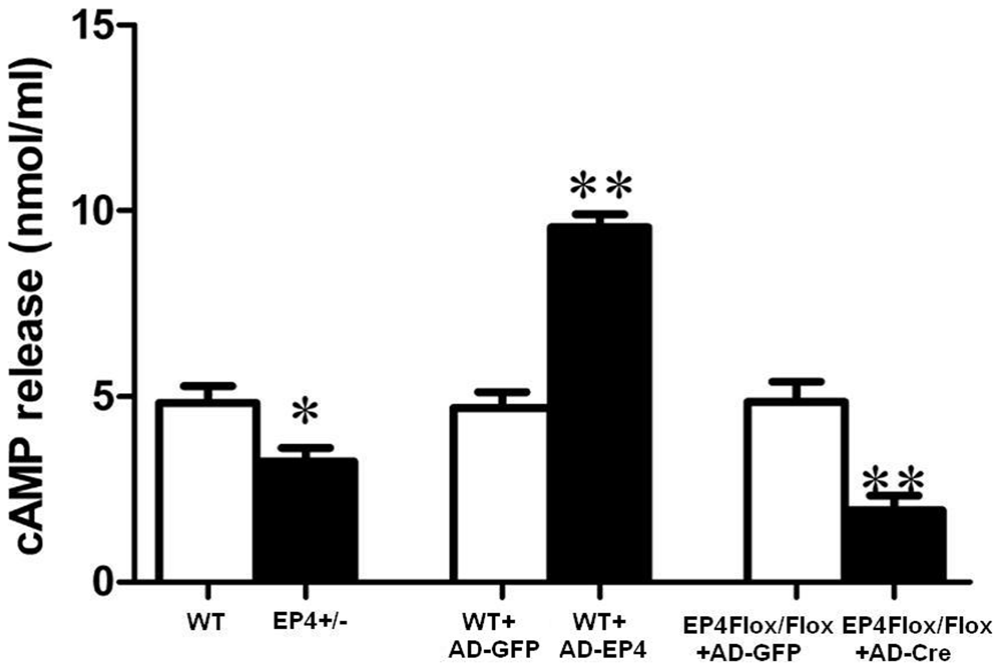
EP4 deficiency reduced cAMP secretion. Effect of cAMP release from GMCs in up or down regulation of EP4 expression. Concentration of cAMP in cell supernatants were measured 24 h after infection of AD-Cre (MOI = 10) or AD-EP4 (MOI = 5). (*P<0.05, **P<0.01 vs Control group, the means and error bars are the result of biological replicates).

The down and up regulation of EP4 receptor in GMCs was confirmed by measuring the cAMP production. Activation of EP4 receptor results in stimulation of adenylyl cyclase and increases intracellular cAMP. As shown in Fig. 3, in contrast to WT GMCs, cAMP production was decreased in EP4^+/−^ GMCs, suggesting functional EP4 receptor deletion in GMCs of EP4^+/−^ mice. Furthermore, cAMP production is additively increased when WT GMCs were transfected with AD-EP4 and significantly decreased when EP4^Flox/Flox^ GMCs were transfected with AD-Cre.

### The effect of EP4 on accumulation of extracellular matrix in mouse GMCs

**Figure 4:**
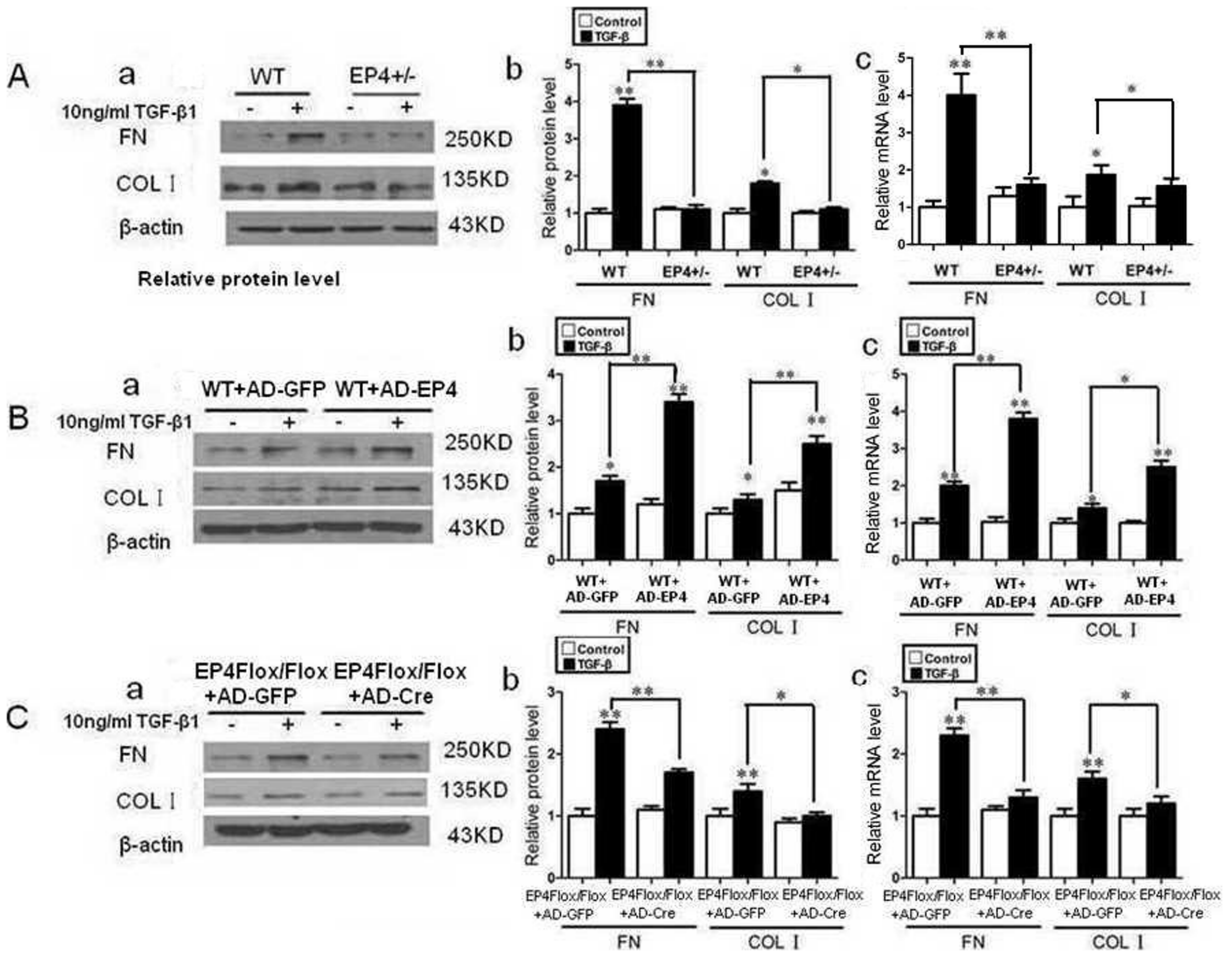
The effect of EP4 on accumulation of extracellular matrix in mouse GMCs. A**:** The WT and EP4^+/−^ mice GMCs were stimulated by 10 ng/ml TGF-β1 for 24 h. The expressions of FN and Col I protein were assessed by Western Blot. B: WT mice GMCs infected with AD-EP4 and AD-GFP were stimulated by 10 ng/ml TGF-β1 for 24 h. The expressions of FN and Col I protein were assessed by Western Blot. C: EP4^Flox/Flox^ mice GMCs infected with AD-Cre and AD-GFP were stimulated by 10 ng/ml TGF-β1 for 24 h. The expressions of FN and Col I protein were assessed by Western Blot. (a: Western Blot; b: Quantification of FN and Col I expression is achieved using densitometric values normalized to β-actin levels; c: The expressions of FN and Col I mRNA were assessed by RT-PCR. (*P<0.05, **P<0.01 vs Control group, the means and error bars are the result of biological replicates).

The accumulation of glomerular ECM is one of the critical pathological characteristics of renal diseases. FN and Col I are important constituents of ECM. The expressions of FN, Col I were assessed by Western Blot and RT-PCR. According to our previous experiments, the functional activity of TGF-β1 was most effective at a concentration of 10 ng/ml, this concentration was chosen for subsequent experiments. After the mice GMCs were stimulated by 10 ng/ml TGF-β1 for 24 h, the expressions of FN, Col I protein of EP4^+/−^ and WT mice GMCs as well as adenovirus infected EP4^Flox/Flox^ GMCs were both increased compared with untreated GMCs (P<0.05). The expressions of FN and Col I protein of EP4^+/−^ GMCs were decreased significantly compared with those of WT mouse GMCs (P<0.05) (Fig. 4A). WT mouse GMCs infected with AD-EP4 are stimulated with 10 ng/ml TGF-β1 for 24 h, inducing a significant increase in FN, Col I expression compared with those of TGF-β1 treated AD-GFP infected controls (P<0.05) (Fig. 4B). EP4^Flox/Flox^ mice GMCs infected with AD-Cre expressed lower level of FN, Col I protein than those of AD-GFP infected controls (P<0.05) (Fig. 4C). The results obtained by RT-PCR were consistent with those obtained with Western blot. The results showed that the expression of FN and Col I changed with the expression of EP4, in that they were increased with over expression of EP4 and decreased with down regulation of EP4.

### EP4 deficiency decreased the expression of enzymes involved in the TGF-β1-induced synthesis of PGE2

**Figure 5:**
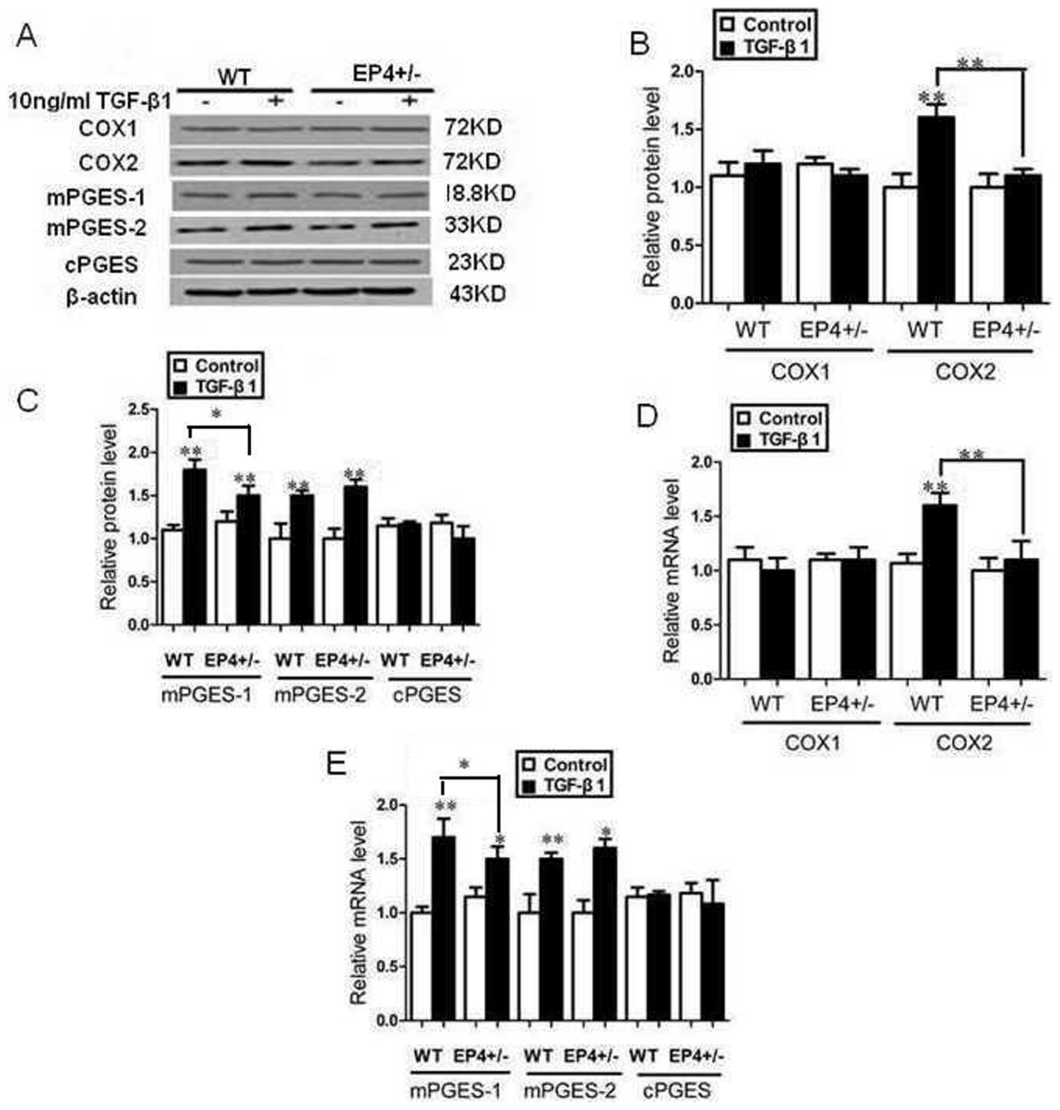
EP4 deficiency decreased the expression of enzymes involved in the TGF-β1-induced synthesis of PGE2. A, B, C: The WT and EP4^+/−^ mice GMCs were stimulated by 10 ng/ml TGF-β1 for 24 h. The expressions of COX1, COX2, mPGES-1, mPGES-2 and cPGE were assessed by Western Blot. (A: Western Blot; B, C: Quantification of COX1, COX2, mPGES-1, mPGES-2 and cPGE expression is achieved using densitometric values normalized to β-actin levels); D, E: The expressions of COX1, COX2, mPGES-1, mPGES-2 and cPGE were assessed by RT-PCR. (*P<0.05, **P<0.01 vs Control group, the means and error bars are the result of biological replicates).

**Figure 6:**
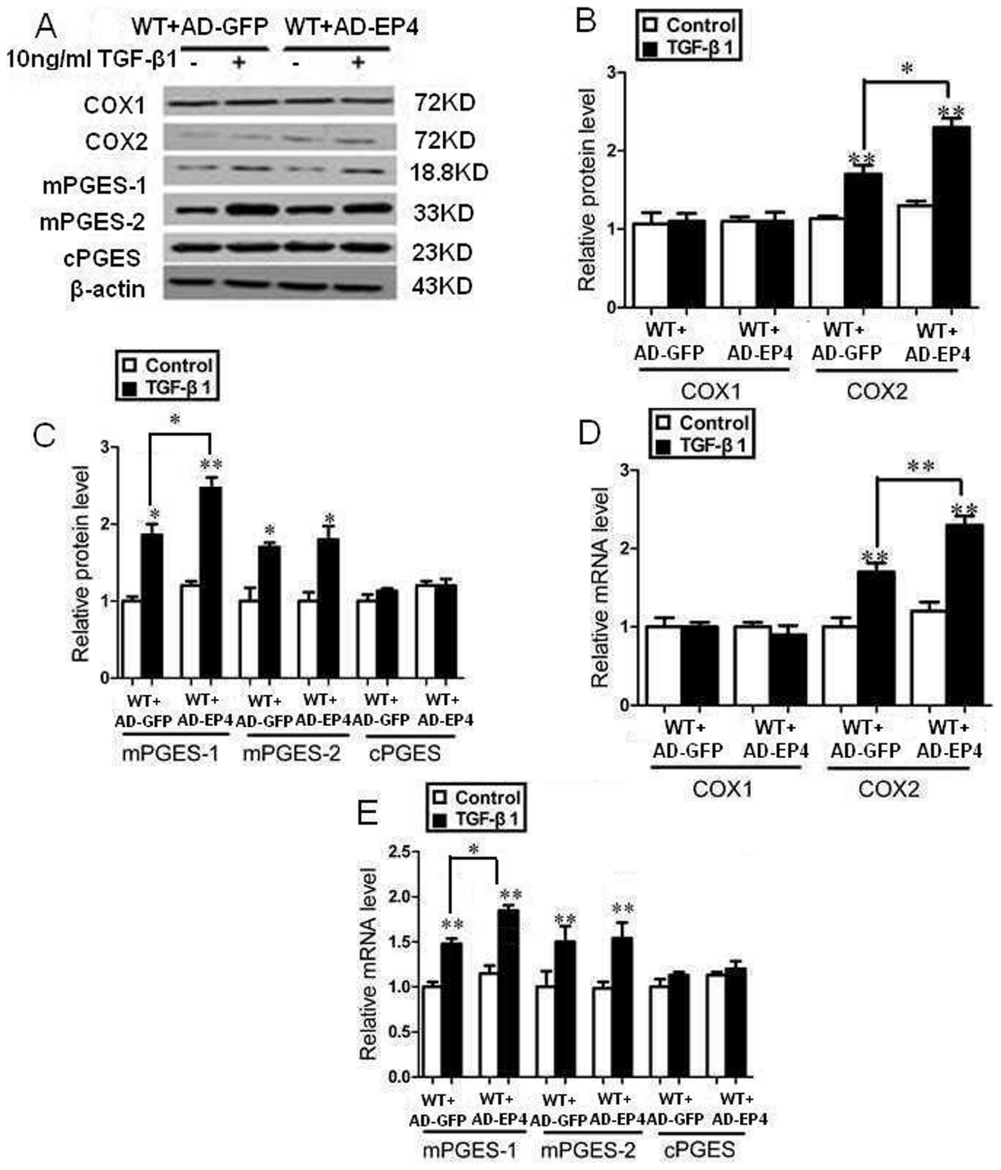
EP4 up-regulation increased the expression of enzymes involved in the TGF-β1-induced synthesis of PGE2. A, B, C: The AD-EP4 and AD-GFP infected WT mice GMCs were stimulated by 10 ng/ml TGF-β1 for 24 h. The expressions of COX1, COX2, mPGES-1, mPGES-2 and cPGE were assessed by Western Blot. (A: Western Blot; B, C: Quantification of COX1, COX2, mPGES-1, mPGES-2 and cPGE expression is achieved using densitometric values normalized to β-actin levels); D, E: The expressions of COX1, COX2, mPGES-1, mPGES-2 and cPGE were assessed by RT-PCR. (*P<0.05, **P<0.01 vs Control group, the means and error bars are the result of biological replicates).

**Figure 7:**
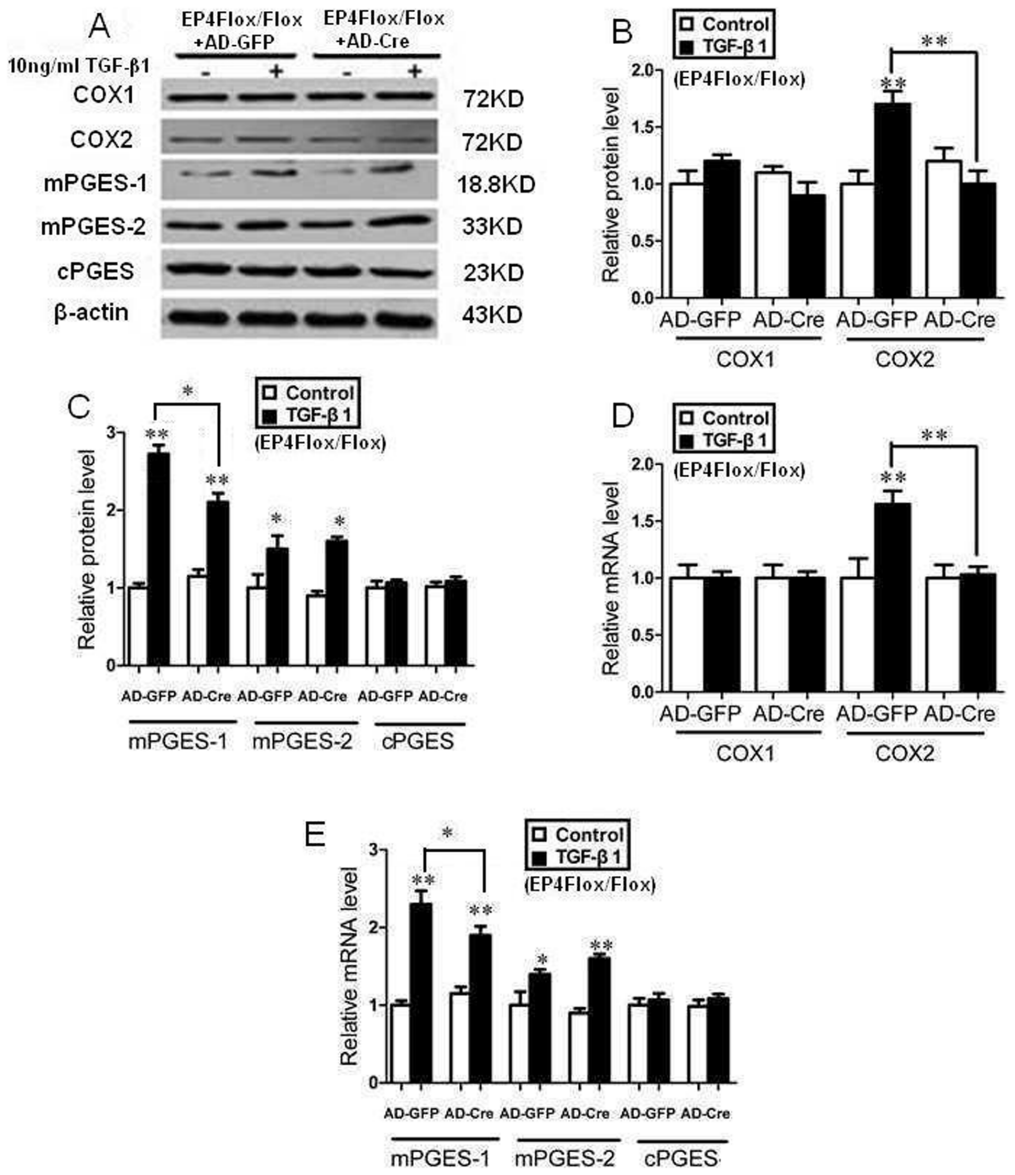
EP4 silencing decreased the expression of enzymes involved in the TGF-β1-induced synthesis of PGE2. A, B, C: The AD-Cre and AD-GFP infected EP4^Flox/Flox^ mice GMCs were stimulated by 10 ng/ml TGF-β1 for 24 h. The expressions of COX1, COX2, mPGES-1, mPGES-2 and cPGE were assessed by Western Blot. (A: Western Blot; B, C: Quantification of COX1, COX2, mPGES-1, mPGES-2 and cPGE expression is achieved using densitometric values normalized to β-actin levels); D, E: The expressions of COX1, COX2, mPGES-1, mPGES-2 and cPGE were assessed by RT-PCR. (*P<0.05, **P<0.01 vs Control group, the means and error bars are the result of biological replicates).

The expressions of COX1, COX2, mPGES-1, mPGES-2 and cPGES were assessed by Western Blot and RT-PCR. After the mouse GMCs were stimulated by 10 ng/ml TGF-β1 for 24 h, the expression of COX2, mPGES-1, mPGES-2 protein and mRNA were significantly increased compared with TGF-β1 untreated groups, the expression of COX1, cPGES mRNA and protein had no significant difference among all trial groups (P>0.05, Fig. 5, 6, 7).

The expressions of COX2 and mPGES-1 protein and mRNA in EP4^+/−^+TGF-β1 and EP4^Flox/Flox^ + AD-Cre+TGF-β1 groups decreased significantly than those of WT+TGF-β1 and EP4^Flox/Flox^ + AD-GFP+TGF-β1 groups respectively. WT+AD-EP4+TGF-β1 group had significantly higher COX2 and mPGES-1 protein and mRNA expressions than those of WT+AD-GFP+TGF-β1 group. In addition, there was no significant difference in expression of COX1, mPGES-2 and cPGES protein and mRNA between the three groups and their controls (P>0.05) (Fig. 5, 6, 7).

### PGE2 production regulated by EP4

**Figure 8:**
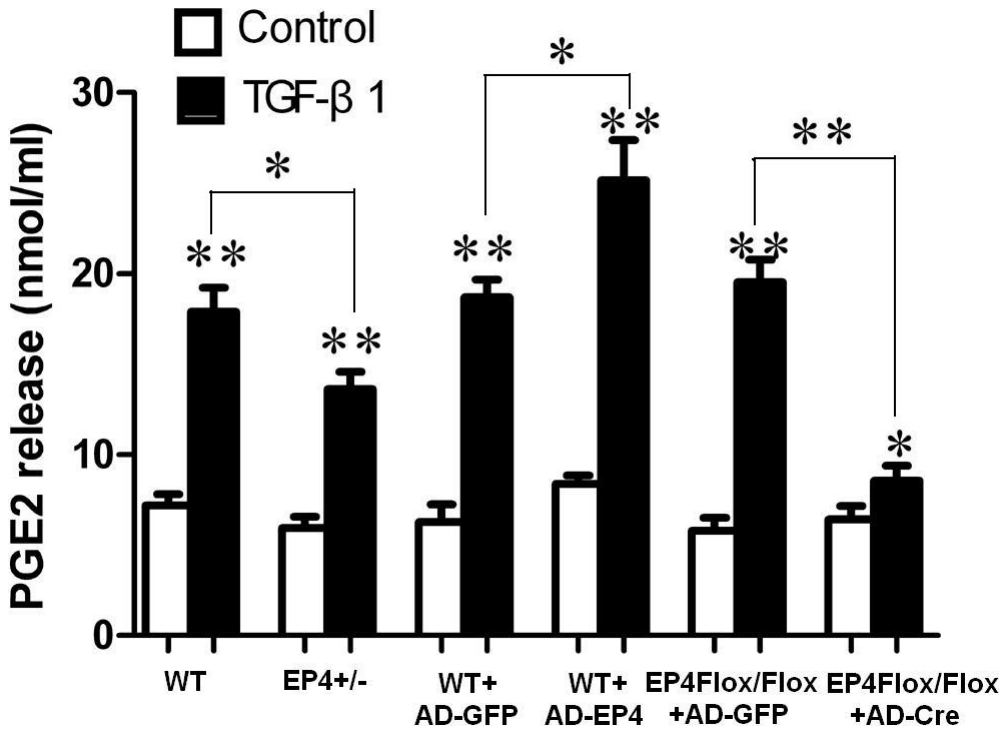
PGE2 Elisa analysis. PGE_2_ levels in the cell supernatant of WT, EP4+/−, AD-GFP, AD-EP4, AD-GFP and AD-Cre mouse GMCs with or without TGF-β1 treatment. The values are means±standard deviations, the means and error bars are the result of biological replicates, *P<0.05, **P<0.01 vs Control group.

As shown in Fig. 8, TGF-β1 treatment significantly increased PGE2 synthesis compared with their controls. TGF-β1 treatment significantly increased PGE2 synthesis in WT and AD-EP4 transfected WT GMCs compared with EP4^+/−^ and AD-GFP transfected WT GMCs respectively, AD-Cre transfected EP4^Flox/Flox^ GMCs had significantly reduced PGE2 production compared with AD-GFP transfected EP4^Flox/Flox^ GMCs. Collectively, these results suggests that EP4 plays a critical role in TGF-β1-induced PGE2 production.

### Biochemical indices of renal fibrosis

**Figure 9:**
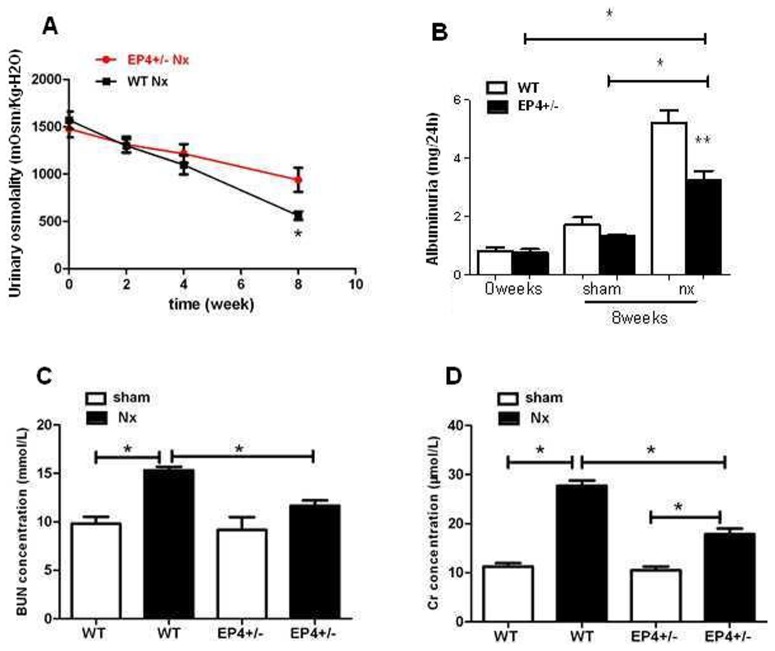
Measurement of renal function. Effect of EP4 on (A)Urinary osmolality, (B)Albuminuria, (C)Blood BUN and (D)Cr expression in the kidneys of 5/6 Nx mice. *P<0.05, **P<0.01.

The urinary osmolality at 8 week of 5/6 Nx mice were decreased from 1516.23±96.51 to 552.79±72.52 mOsm/kg·H_2_O in WT Nx (n = 10 for each time point) and from 1465.90±97.31 to 934.32±64.69 mOsm/kg·H_2_O in EP4^+/−^ Nx mice (n = 10 for each time point) (Fig. 9A). The albuminuria in WT Nx (5.24±0.27 mg/24 h) and EP4^+/−^Nx mice (3.56±0.18 mg/24 h) were significantly increased compareing with WT (1.93±0.12 mg/24 h) and EP4^+/−^ sham group (1.72±0.09 mg/24 h) at 8 weeks after surgery and WT Nx group has much higher albuminuria than EP4^+/−^ Nx group (Fig. 9B). Plasma BUN level in 5/6 Nx mice (WT: 15.78±0.87; EP4^+/−^: 12.45±0.67 mmol/L) was significantly higher than those in shams (WT: 10.21±0.51; EP4^+/−^: 9.87±0.47 mmol/L). Plasma Cr concentration in 5/6 Nx mice (WT: 28.78±1.47; EP4^+/−^: 17.52±1.32 µmol/L) was dramatically higher than those in shams (WT: 10.17±0.41; EP4^+/−^: 10.04±0.53 µmol/L). The data showed there was a significant increase of the Cr and BUN levels in the WT 5/6 Nx mice compared to the EP4^+/−^ Nx mice at 8 weeks after surgery (Fig. 9C & D).

### The effect of EP4 receptor deletion from the GMCs on the renal fibrosis disease

**Figure 10:**
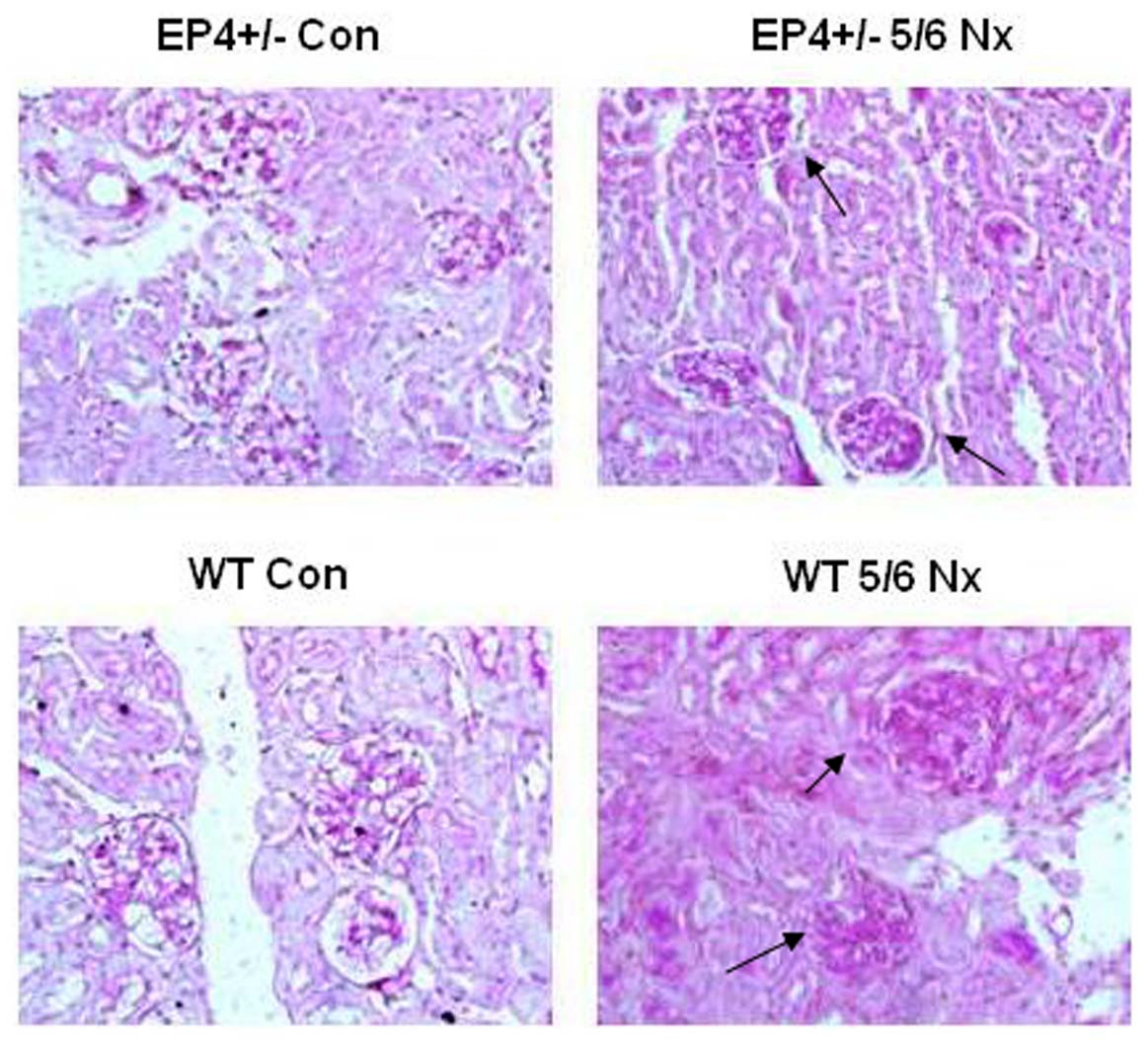
Acid-Schiff staining. Milder renal pathology is observed in 5/6Nx EP4^+/−^ mice. Kidneys are removed and disease pathology is visualized by periodic acid–Schiff staining of paraffin-embedded sections at 8 weeks after 5/6 Nx. Milder glomerular pathology is observed in 5/6 Nx EP4^+/−^ mice with less matrix deposition than 5/6 Nx WT mice. Magnification,400.

**Figure 11:**
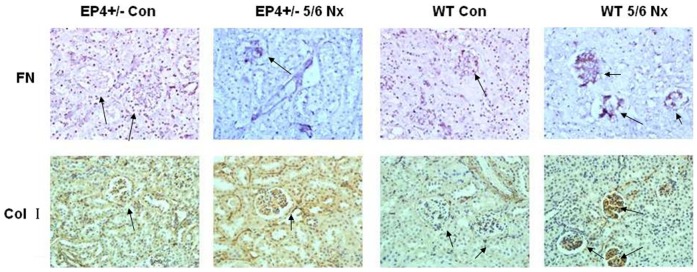
Immunohistochemistry. Glomerular FN and Col I immunostaining were markedly lower in EP4^+/−^5/6 Nx than in the WT 5/6 Nx groups and the number of glomerular cells positively immunostaining for the FN and Col I were similarly increased in EP4^+/−^5/6 Nx and WT 5/6 Nx mouse relative to sham animals. The integrated optical density of the positive material in each glomerulus and the glomerular area were measured by the morphological analysis system. Magnification,400.

We investigated the impact of EP4 receptor deletion from the GMCs on renal fibrosis. For this purpose, groups of EP4^+/−^ mice underwent 5/6 nephrectomy. Eight weeks later, representative morphological changes in kidney tissues at the end of the study were visualized by periodic acid–Schiff staining of paraffin-embedded sections as shown in Fig. 10. Glomerular pathology of 5/6 nephrectomy EP4^+/−^ mice exhibited qualitatively milder glomerular lesions, with less matrix deposition than their 5/6 nephrectomy WT littermate controls. A marked accumulation of ECM in glomeruli of the 5/6 nephrectomy WT mice group was observed. Immunohistochemical staining was used to examine the effect of EP4 on the FN and Col I expression in the glomeruli of 5/6 nephrectomy mice. As demonstrated in Fig. 11, renal glomeruli of WT and EP4^+/−^ control group express trivial amount of FN and Col I. However, compared with the control group, the expression of FN and Col I in the renal glomeruli was significantly enhanced in the 5/6 nephrectomy group (P<0.01), indicating extracellular matrix accumulation. Meanwhile, FN and Col I expression in glomeruli increased more significantly in the WT 5/6 nephrectomy group compared with those of EP4^+/−^5/6 nephrectomy group (P<0.05). The ratio of the integrated optical density of FN and Col I in renal glomerulus and the glomerular area confirmed this result (Table 2, 3). This result indicated that EP4 receptor deficiency could inhibit FN and Col I expression in the renal glomeruli of 5/6 nephrectomy mice, suppress accumulation of the extracellular matrix and prevent development of renal fibrosis.

**Table 2:** Relative content of FN in the renal glomeruli.

Group	Integrated optical density	Glomerular area (µm^2^)	Ratio
WT Con	13.12±3.77	3732.40±419.39	3.52±2.55
WT 5/6Nx	346.39±87.38	4280.43±523.72	81.92±33.59 aa
EP4^+/−^ Con	22.16±12.39	3123.87±313.04	6.87±4.75
EP4^+/−^5/6Nx	153.46±48.29	2745.76±280.80	53.93±23.12 ^bbc^

Compared with WT Con group,

a P<0.05,

aa P<0.01, compared with EP4^+/−^ Con group,

b P<0.05,

bb P<0.01, compared with WT 5/6Nx group,

c P<0.05,

cc P<0.01.

**Table 3:** Relative content of Col I in the renal glomeruli.

Group	Integrated optical density	Glomerular area (µm^2^)	Ratio
WT Con	22.36±6.13	3935.23±559.25	5.68±2.89
WT 5/6Nx	379.20±74.19	4587.13±683.52	82.67±39.76 aa
EP4^+/−^ Con	29.79±16.46	3577.83±913.12	8.33±4.96
EP4^+/−^5/6Nx	126.66±23.12	3449.39±640.26	36.72±16.30 ^bbc^

Compared with WT Con group,

a P<0.05,

aa P<0.01, compared with EP4^+/−^ Con group,

b P<0.05,

bb P<0.01, compared with WT 5/6Nx group,

c P<0.05,

cc P<0.01.

## Discussion

Renal fibrosis is characterized by a progressive substitution of cellular elements by ECM proteins. It is caused by an imbalance between proliferation, necrosis, and apoptosis of cells [16], as well as the loss of normal equilibrium between synthesis and degradation of ECM proteins [17], [18]. GMCs seem to play a key role in the genesis of glomerular sclerosis [19]. Studies have demonstrated that all four known EP receptor subtypes are expressed in GMCs [20]. PGE2 exerts various observable effects on controlling human kidney functions, which are related to the distribution of these four receptor proteins [21]. In this regard, we investigated the function of EP receptors in GMCs to further understand their roles in the process of renal fibrosis.

TGF-β has been recognized as a central player in many pathological events related to CKD progression, at the glomerular, tubulointerstitial and vascular levels. The experimental inhibition of TGF-β has been shown to reverse the chronic renal damage in a number of different pathological scenarios [21], [22], on the contrary, overexpression of TGF-β can cause renal fibrosis. In the current study, we selected 10µg/L of TGF-β1 as the stimulator to induce the accumulation of ECM in mesangial cells. FN and Col I were analyzed as a marker of ECM accumulation in TGF-β1-treated mice GMCs. Our results showed that the expression levels of FN, Col I protein and mRNA in WT+TGF-β1 group were significantly higher than those of WT group. Our results demonstrated that continuous stimulation of TGF-β1 led to ECM accumulation in mesangial cells, which in turn led to glomerulosclerosis.

Recent studies by Vukicevic et al [23], showed that EP4 played an important role in renal fibrosis. In animal experiments, the endogenous PGE2-EP4 system of tubular epithelium limited the development of fibrosis by suppressing inflammatory responses. Furthermore, PGE2 plays an important role in acute renal failure via the EP4 receptor. Both EP2 and EP4 receptors are equally important in preventing the progression of chronic kidney failure. Stitt-Cavanaqh et al [15] showed that podocyte-specific overexpression of desensitization-resistant EP4 resulted in more severe renal injury and lower survival rate in 5/6 nephrectomy model, whereas, podocyte-specific EP4 deletion significantly relieved renal injury. If such different effects of EP4 in glomeruli versus interstitial cells were confirmed by other studies, the use of EP4 receptor agonists could be limited in progressive glomerular diseases.

In the present study, we hypothesized that expression of EP4 in mouse GMCs could play a role in the pathophysiology of renal fibrosis, and we designed experiments to analyze the effects of EP4 in mesangial ECM accumulation. We generated EP4^+/−^ and EP4^Flox/Flox^ mice and established primary cultures of WT, EP4^+/−^ and EP4^Flox/Flox^ mouse GMCs. After stimulating mouse GMCs with 10 ng/ml TGF-β1 for 24 h, the FN, Col I mRNA and protein levels in the EP4^+/−^ +TGF-β1 group were significantly lower than those in the WT+TGF-β1 group. In addition, we found that overexpression of EP4 in WT mouse GMCs led to higher expression of FN, Col I protein and mRNA, whereas EP4 silencing in EP4^Flox/Flox^ mouse GMCs resulted in less expression of FN, Col I protein and mRNA induced by TGF-β1 than those in control group. Thus, we found that upregulation of EP4 could promote TGF-β1-induced ECM accumulation in mouse mesangial cells. Similar results were observed in experiments in vivo. Compared with 5/6 nephrectomy WT mice, the expressions of FN and Col I in glomeruli were significantly reduced in the 5/6 nephrectomy EP4^+/−^ mice. Based on the results from in vitro and in vivo observations, we concluded that conditional deletion of this prostanoid receptor subtype from GMCs confered partial protection from such glomerular damage. In addition, albuminuria, BUN and Cr concentration in WT 5/6 Nx mice were significantly higher than those in EP4^+/−^5/6 Nx mice at 8 weeks after surgery while urinary osmolality was decreased. Our findings therefore support a maladaptive role for the PGE2 EP4 receptor in the context of renal fibrosis.

Previous studies showed that podocyte-specific overexpression of COX-2 in mice made them more susceptible to glomerular injury in models of minimal change disease [24], [25]. Furthermore, glomerular PGE2 levels reduced and were negatively correlated with proteinuria and glomerular sclerosis in subtotally nephrectomized rats treated with a selective COX2 inhibitor [26]. The fact that COX2 inhibitor can reduce the proliferation of GMCs and inhibit the progress of glomerular fibrosis suggests that COX2 may participate in the regulation of GMCs proliferation. In vitro studies have demonstrated that PGE2 can regulate renal epithelial regeneration via EP4 receptor by inhibiting apoptosis and epithelial-mesenchymal transition [27]. PGE2 can also induce COX2 expression in cultured mouse podocytes. Specifically, it is not the Gq-coupled EP1 subtype, but the G protein-coupled EP4 receptor, which mediates PGE2-induced COX2 expression in conditionally immortalized mouse podocytes [13]. mPGES-1 and COX2 play a coupling role in the pathology and progression of chronic renal injury and their expression is highly inducible in response to inflammatory stimuli with production of PGE2 [28]. This raises the possibility that the COX2-PGE2-EP4 system could mediate the renal fibrosis and contribute to mesangial ECM accumulation. We showed here that the expression of EP4 was selectively targeted by TGF-β1 in WT, EP4^+/−^ and EP4^Flox/Flox^ GMCs, and more important, that EP4 promoted the activities of COX2, mPGES-1 and PGE2 (Figure 5, 6, 7, 8). The results of these EP4 activities suggested an important function of EP4 signaling in promoting the early stages of renal fibrosis. Another striking finding of this study was that the complete pathological redundancy existed among FN, Col I and EP4, and promoted renal fibrosis and progression (Figure 10, 11). Thus, our findings provide the evidence that targeting EP4 receptor may upregulate the expression of mPGES-1 and COX2 in the renal tissue, and lead to the proliferation of GMCs by participating in the inflammation. COX2 overexpression increases PGE2 expression in inflammation areas, while PGE2 as an inflammatory mediator leads to accumulation of more inflammatory cells and aggravates the inflammation eventually. This sets up a vicious circle of increasing TGF-β1-induced GMCs ECM accumulation.

EP2 and EP4 are both in the family of G-protein coupled receptors and both receptors were initially characterized as coupling to G proteins and increasing intracellular cAMP formation. For this reason, we cannot rule out the possibility that the EP2 receptor also contributes to renal fibrosis development. The specificity of EP2 *vs* EP4 receptors to affect TGF-β signaling way also reflect differences in their ability to internalize in response to PGE2 stimulation, as well as in their preferential coupling to cAMP-PKA. Further studies are needed to determine the roles of the EP2 receptor in the renal fibrosis and the downstream effectors and genes targeted by the COX2/PGE2/EP4/cAMP signaling axis and to elucidate how these elements actively affect the function of TGF-β in the development and progression of renal fibrosis.

In summary, we have shown that EP4 signaling is responsible for the effects of PGE2 on ECM accumulation in GMCs and our finding reveal a positive feedback loop between COX2 and PGE2 mediated by the EP4 receptor. It suggests that EP4 may have many effects in different parts of the kidney and also in renal fibrosis progression. Thus selective chemotherapeutic targeting of EP4 may provide new alternatives to alleviate the renal fibrosis.
